# The Evolution of Human Basophil Biology from Neglect towards Understanding of Their Immune Functions

**DOI:** 10.1155/2016/8232830

**Published:** 2016-12-19

**Authors:** Markus Steiner, Sara Huber, Andrea Harrer, Martin Himly

**Affiliations:** ^1^Division of Allergy & Immunology, Department of Molecular Biology, University of Salzburg, Salzburg, Austria; ^2^Laboratory for Immunological & Molecular Cancer Research, Paracelsus Medical University, Salzburg, Austria; ^3^Department of Neurology, Paracelsus Medical University, Salzburg, Austria

## Abstract

Being discovered long ago basophils have been neglected for more than a century. During the past decade evidence emerged that basophils share features of innate and adaptive immunity. Nowadays, basophils are best known for their striking effector role in the allergic reaction. They hence have been used for establishing new diagnostic tests and therapeutic approaches and for characterizing natural and recombinant allergens as well as hypoallergens, which display lower or diminished IgE-binding activity. However, it was a long way from discovery in 1879 until identification of their function in hypersensitivity reactions, including adverse drug reactions. Starting with a historical background, this review highlights the modern view on basophil biology.

## 1. Introduction

Basophils are well known for their allergic effector function, a feature applied in the basophil activation test. According to current literature basophils are additionally conceived in the center between the innate and adaptive immune responses [1, 2]. The following chapter intends to review the most important findings during the past 150 years of basophil biology. 

*A Historical Perspective: from Discovery to Recognition as Allergic Effector Cell*. In 1879 Ehrlich discovered a cell type with dark blue granula in peripheral blood staining experiments with basic dyes [3, 4], which he named “basophilic granulocytes” or “basophils” according to their unique appearance. He previously had described human mast cells in his doctoral thesis in 1878 [5], thus initially referred to basophils as mast leucocytes or blood mast cells. Shortly thereafter it was found out that they were leucocytes of myeloid origin [6]. In 1894 and 1895, Kanthack and Hardy continued to define the appearance and morphology of mast cells and basophils [7]. They characterized the latter as “*the finely granular basophil *cell” appearing solely in the blood which was surprisingly accurate. Moreover, they were the first to describe basophils as very unstable and recognized their “explosive nature,” in response to “certain chemical stimuli.” This very basic understanding of basophil biology remained state of knowledge for almost 60 years until the 1950s which may have been due to their low abundance. A significant role of basophils was questioned which pushed them into the position of an “underdog” [8–11].

During this time, however, descriptions about hypersensitivity reactions in patients emerged including research efforts to elucidate their etiology. Portier and Richet were the first mentioning the term anaphylaxis in 1902 when they found out that dogs became hypersensitive to anemone toxins after repeated injections [12] and Pirquet et al. coined the term “allergy” to describe the immune response in hypersensitivity reactions [13–15]. With anaphylatoxin discovered in the serum [16] and histamine shown to be involved in smooth muscle contraction [17] different molecules were proposed as key candidates responsible for such reactions. The following two studies showed that anaphylaxis-triggering molecules must be contained in the blood or serum: Ramirez reported on a case of transmitted hypersensitivity in 1919 when a person got an asthmatic attack after receiving a blood transfusion but had so far no history of hypersensitivity [18]. A similar discovery was made by Prausnitz and Küstner in 1921 [19], when Küstner, who was allergic to fish, locally transferred his allergy to Prausnitz's skin. The molecules responsible for the reaction, however, were not yet identified. Further on prostaglandins and leukotrienes, slow-reacting substances, with a late reaction onset, were discovered by von Euler et al. in the 1930s [20, 21].

It was in 1934 that a delayed reaction in the skin after repeated injection of a foreign protein was linked to basophils and described as the so-called cutaneous basophil hypersensitivity by Jones and Mote [22]. Five years later, Trager described a similar basophil accumulation in the setting of a tick bite and stated that basophils are crucial for the induction of immune reactions. He claimed that the histamine-induced itching was essential for the detection and removal of the tick [23].

Despite these early reports it took almost 20 years until discoveries in hypersensitivity were linked to basophils and mast cells. It was in 1953 and 1955 when Riley et al. showed that histamine was stored predominantly in basophil granules and suggested that these cells were involved in anaphylaxis [24, 25]. Shelley and Juhlin were the ones who established a test system for detecting histamine release from basophils; the basophil activation test was born. The principle of the first basophil activation tests was based on degranulation monitoring using optical microscopy. Whole blood or leukocytes (enriched by filtration) were subjected to activation stimuli followed by rapid liquid fixation and staining with basic dyes. Reduced levels of granular staining indicated an activation-induced degranulation [26, 27]. Furthermore, Juhlin and Shelley proved that histamine was released from mast cells and basophils [28] by a radio immune assay which was based on binding competition to an anti-histamine antibody between cell-derived histamine in the sample and ^125^l-labeled histamine. The detailed degranulation mechanism of basophils was finally explained when Ishizaka and Bennich independently identified the serum factor responsible for this immediate reaction in the years from 1966 to 1968 which they agreed to call immunoglobulin (Ig) E. Their experiments were based on the Prausnitz-Küstner reaction (for safety reasons not recommended as involving intradermal serum transfer from an atopic patient to a nonallergic control person) which was blocked by antiserum from fractionated serum of allergic individuals or by myeloma-derived IgE [29–31]. Some years later Ishizaka et al. identified receptors for IgE on basophils and mast cells. He described that binding of anti-IgE and receptor aggregation led to the release of histamine from these cells [32–34]. Soon after, Kulczycki Jr. et al. showed that IgE was bound with high affinity, and Jarrett and Bazin found out that helminthic parasite infection was the most powerful inducer of IgE. They showed that rats infected with parasites had a strong elevation in their total serum IgE levels [35, 36].

Measuring leukotrienes from supernatants of stimulated basophils by radio immune-assay as alternative to histamine release was pointed out by MacGlashan Jr. et al. in 1986 [37]. Basophil counting and activation monitoring by flow cytometry finally came up in the 1990s when Knol et al. started to utilize activation-induced and degranulation-associated changes on the cell surface. They monitored the appearance of CD63, alias Lamp-3, expressed in intracellular granule membranes, with a fluorescence-labeled monoclonal anti-CD63 detection antibody [38]. The CD63 molecule was previously cloned and analyzed by Metzelaar et al. [39]. The studies by Gibbs et al. in 1996 on IL-4 and IL-13 secretion from basophils [40] were a further milestone for determining the immunological capabilities of basophils. One year later, the chemokine receptor, CCR3, was described to be highly expressed on human blood basophils [41]. Bühring et al. added another important basophil-specific surface antigen in 1999, when he discovered the ectoenzyme CD203c, a versatile marker for basophil identification and activation [42]. In the same year basogranulin was described for the first time following the development and characterization of a basophil-specific antibody, the anti-basogranulin antibody BB1 [43]. Hirai et al. proposed another identification option for basophils for flow cytometry by gating cells with low side scattering which are prostaglandin DP2 receptor CRTH2 (CD294)-positive and CD3-negative [44]. Further activation markers (CD13, CD107a, and CD164) were identified by Hennersdorf et al. [45].

**Figure 1:**
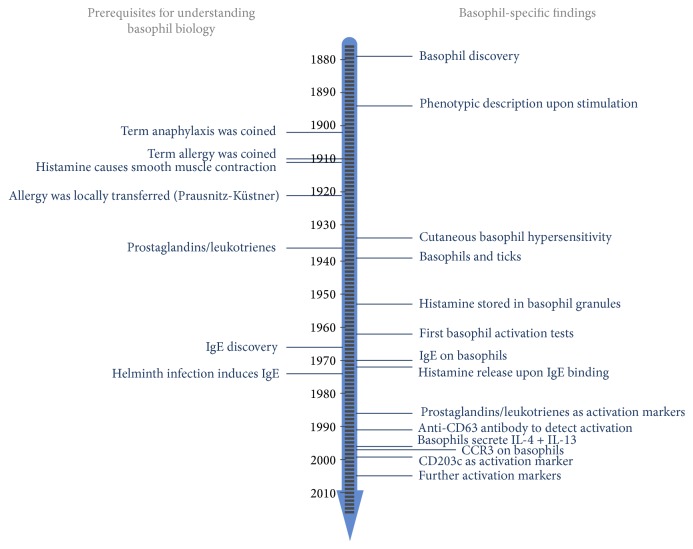
Timeline of discoveries during the evolution of basophil research.

Summing up, during the last decades, our knowledge on basophils drastically increased with the most relevant findings depicted in the timeline of Figure 1. To date, these new findings are utilized for basic and clinical research questions related to gain a deeper insight into the biology of basophils and to develop advanced diagnostic systems for patients suffering from hypersensitivity reactions.

## 2. Basophil Development

**Figure 2:**
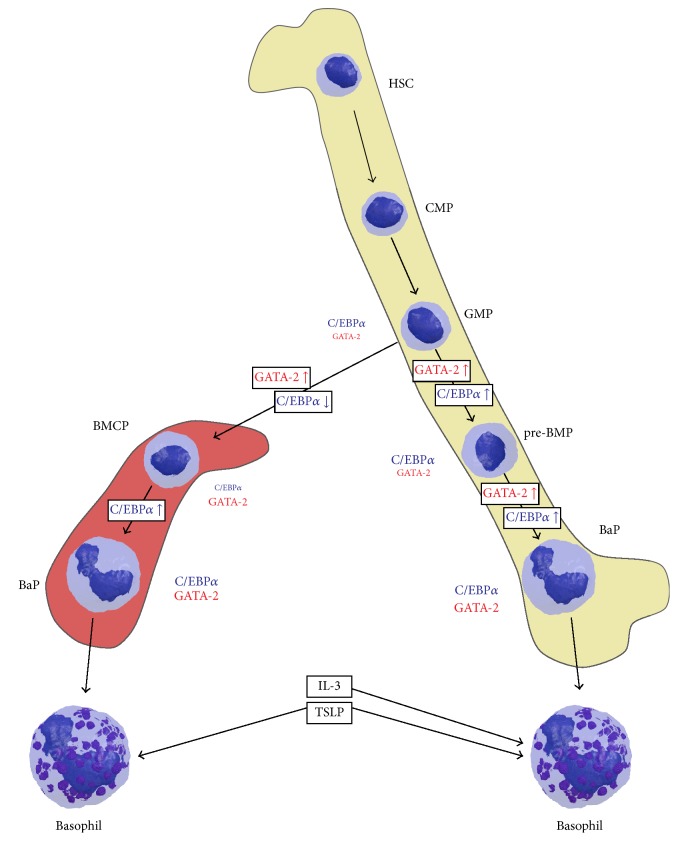
Influence of C/EBP*α* and GATA-2 on the basophil development in spleen (left) and bone marrow (right). In the final maturation step basophils might be either elicited by IL-3 or TSLP. HSC: hematopoietic stem cell; CLP: common lymphoid progenitor; CMP: common myeloid progenitor; GMP: granulocyte/monocyte progenitor; BMCP: basophil/mast cell progenitor; pre-BMP: prebasophil mast cell progenitor; BaP: basophil progenitor; IL-3: interleukin 3; TSLP: thymic stromal lymphopoietin.

Though mature basophils are extensively studied, the hematopoietic origin of these cells is scarcely understood [46]. As reviewed in detail elsewhere [47, 48], findings in mouse blood cell hematopoiesis suggest basophils to develop from hematopoietic stem cells (HSCs)* via* common myeloid progenitors (CMPs), granulocyte-monocyte progenitors (GMPs), and (possibly) granulocyte progenitors (GPs) in the bone marrow [47, 49]. Further development continues in the bone marrow (prebasophil mast cell progenitor, pre-BMC) or in the spleen (basophil mast cell progenitor, BMCPs) (Figure 2) [50, 51]. These cell types further differentiate into basophil progenitors and mature basophils which then are released into the blood stream.

During basophil hematopoiesis several transcription factors play crucial roles in determining the fate of the progenitors towards the fully differentiated basophil. Two central factors in basophil development are the CCAAT enhancer-binding protein alpha (C/EBP*α*) and GATA binding protein 2 (GATA-2) [51, 52]. The GMP shows high C/EBP*α* levels and almost no GATA-2. Upregulation of GATA-2 and simultaneous downregulation of C/EBP*α* directs cell development into the basophil/mast cell progenitor (BMCP) line. Hence, the BMCP has high GATA-2 and intermediate C/EBP*α* levels. A further decrease of C/EBP*α* levels directs the cell fate towards mast cell development. Upregulation of C/EBP*α* expression in BMCPs triggers differentiation into basophil progenitors which show high levels of both C/EBP*α* and GATA-2.

Several other transcription factors upstream of C/EBP*α* and GATA-2 were described. Ikaros family zinc finger protein 1 (IKZF1) was shown to negatively regulate basophil development by inhibition of C/EBP*α* expression [53], whereas STAT5 was shown to enhance basophil development by inducing C/EBP*α* and GATA-2 expression [50, 54]. Interferon regulatory factor 8 (IRF8) was found to act upstream of GATA-2 in Irf8 knockout mice showing reduced levels of basophils. A further target of IRF8 is GATA1 which plays a role in the generation of basophil progenitors and aids the final differentiation step into basophils [55, 56].

Two more factors specifically prime basophils for distinct roles, namely, IL-3 and thymic stromal lymphopoietin (TSLP). Short-term IL-3 treatment of bone marrow-derived cells was shown to direct granulocyte-monocyte progenitors into basophil differentiation. Basophils derived from such an IL-3-induced lineage show high IgE reactivity and, therefore are involved in IgE-mediated acquired immunity [57]. In contrast, basophils derived from progenitors treated with TSLP showed lower responsiveness to IgE/antigen complexes but displayed features of a chronic inflammatory cell response including higher IL-18 and IL-33 receptor expression. These cells are predominantly involved in innate immunity. The balance between basophils derived from either IL-3 or TSLP thus is considered crucial for the type of mediator response [58].

## 3. The Basophil in the Immune Network

**Figure 3:**
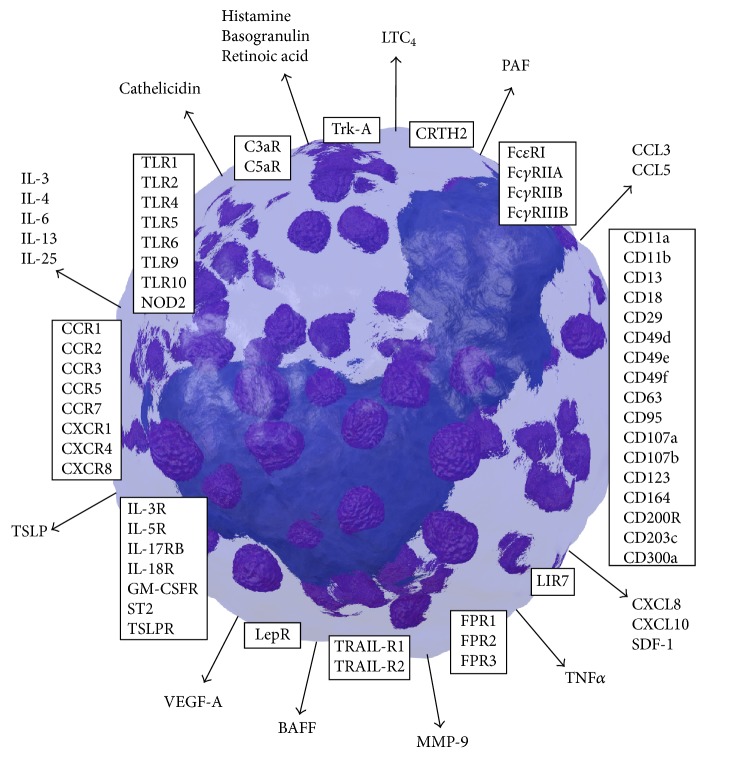
Surface molecules (boxes) and secreted mediators (arrows) of human basophils. BAFF, B cell-activating factor; CxaR, anaphylatoxin receptors; CCL/CXCL, chemokine ligands; CCR, CXCR, chemokine receptors; CD, cluster of differentiation; CRTH2, chemoattractant receptor-homologous molecule expressed on T_H_2 cells; FcxR, immunoglobulin receptors; FPR, formyl peptide receptors; GM-CSFR, granulocyte macrophage colony-stimulating factor receptor; IL, interleukin; IL-R, interleukin receptor; LepR, leptin receptor; LIR, leukocyte immunoglobulin-like receptor; LTC4, leukotriene C4; MMP-9, matrix metallopeptidase; NOD2, nucleotide-binding oligomerization domain-containing protein 2; PAF, platelet activating factor; SDF-1, stromal cell-derived factor 1; ST2, growth stimulation expressed gene 2; TLR, toll-like receptors; TNF*α*, tumor necrosis factor alpha; TRAIL-R, tumor necrosis factor-related apoptosis-inducing ligand receptor; Trk-A, tropomyosin receptor kinase A; TSLP, thymic stromal lymphopoietin; TSLPR, thymic stromal lymphopoietin receptor; VEGF-A, vascular endothelial growth factor A.

Basophil biology and the basophils' interplay with other cells are essentially directed by cytokines, chemokines, and other soluble mediators. In the following chapters important molecules involved in different ways of basophil activation and effector functions, basophil adhesion, migration, and survival, and the dual role of basophils in protection against parasites versus pathogenicity are described. An overview of the most relevant surface molecules and secreted substances is shown in Figure 3.

### 3.1. Basophil Activation

Basophils are best known for their effector function triggered by the release of mediators upon activation. This activation can be mediated by a large number of different molecules such as immunoglobulins, cyto-/chemokines, factors of the complement system, growth factors, bacteria-derived ligands, and proteases interacting with surface receptors (Table 1). The subsequent intracellular signaling pathway leads to release of preformed molecules such as histamine and leukotriene C4 (LTC4), chemotactic factors like the platelet activation factor (PAF) and retinoic acid, basogranulin, cytokines including IL-4, IL-13, IL-6, TNF*α*, and TSLP, chemokines, and antimicrobial peptides. They are all involved in immediate and late-phase reactions of the immune system and symptoms observed in allergic reactions [59, 60].

#### 3.1.1. Activation by Immunoglobulins

**Figure 4:**
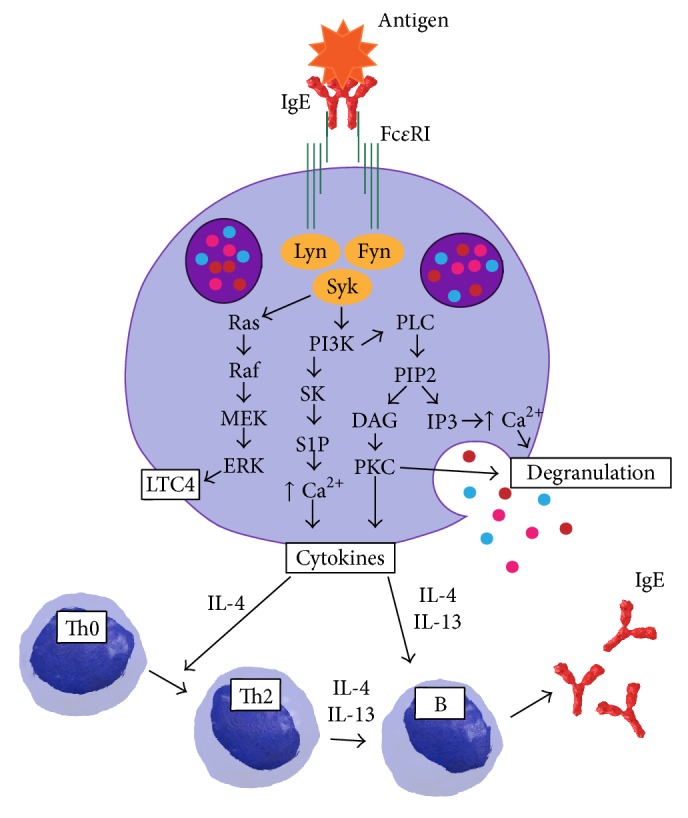
Scheme of basophil activation* via* Fc*ε*RI cross-linking and the involved signaling pathways leading to mediator release. Basophil-derived IL-4 and IL-13 trigger Th2 responses and enhance immunoglobulin production from B cells. DAG, diacylglycerol; ERK, extracellular signal-regulated kinase; Fc*ε*RI, high affinity IgE receptor 1; Fyn, src-related proto-oncogene; IgE, immunoglobulin E; IL, interleukin; IP3, inositol trisphosphate; LTC4, leukotriene C_4_; Lyn, Lck/Yes novel tyrosine kinase; PI3K, phosphoinositide 3-kinase; PIP2, phosphatidylinositol 4,5-bisphosphate; PKC, protein kinase C; PLC, phospholipase C; MEK, MAPK/ERK Kinase; Raf, rapidly accelerated fibrosarcoma; Ras, rat sarcoma; S1P, sphingosine-1-phosphate; SK, sphingosine kinase 1; Syk, spleen associated tyrosine kinase.

*IgE.* IgE/antigen complexes mediate the cross-linking of high affinity IgE receptors Fc*ε*RI on the basophils' surface by downstream signaling of the Src family kinases Fyn, Lyn, and Syk, with activation of the PI3-kinase and the MEK/ERK pathways (Figure 4). It is the most popular and extensively investigated activating mechanism of basophils [61, 62]. As a consequence, intracellular granules fuse with the cell membrane and preformed mediators [63, 64] are released, and granule membrane molecules like CD11b, CD13, CD63, CD107a, CD107b, CD203c, CD200R, CD300a, and the vascular endothelial growth factor A (VEGF-A) [65–67] are translocated to the outer cell surface, the so-called anaphylactic degranulation. Subsequently, mediators like LTC4 are synthesized* de novo* and secreted. During basophil degranulation the two important Th2 response-driving cytokines IL-4 and IL-13 are secreted. The IL-4 secretion is an immediate response with preformed but also newly synthesized (stored RNA) IL-4, whereas IL-13 secretion occurs after several hours of basophil stimulation. IL-4 plays a crucial role in triggering Th2 cell differentiation from naïve CD4^+^ T cells and suppresses the harmful Th1 responses [40, 68, 69]. Basophil-derived IL-4 together with IL-6 activates B cells in humoral protective pathogen responses by enhancing their proliferation and immunoglobulin production [70]. Basophil-derived IL-4 also contributes to the upregulation of the C-C chemokine ligand (CCL)11 in fibroblasts, enhancing eosinophil migration [71], to the differentiation of monocytes into M2 macrophages, and to the proliferation and IL-5 production of group 2 innate lymphoid cells [72, 73].

*IgD*. IgD binds to a so far not further described calcium-mobilizing receptor on basophils and, upon receptor cross-linking, induces antimicrobial, opsonizing, proinflammatory, B cell-stimulatory, and Th2-driving programs, for example, by upregulation of IL-4, IL-13, B cell-activating factor (BAFF), proliferation-inducing ligand (APRIL), CXCL8, CXCL10, and cathelicidin, but has no influence on histamine release [74].

*Secretory IgA*. Secretory IgA was shown to provoke basophil activation by histamine and LTC4 release experiments. Activation of the cells was solely observed upon stimulating IL-3 primed basophils with sepharose-immobilized secretory IgA [75]. The effect was potentiated when secretory IgA was immobilized on plastic plates instead of sepharose beads [76].

*IgG*. Basophils express the IgG receptors Fc*γ*RIIA, Fc*γ*RIIB, and Fc*γ*RIIIB [77–79]. Interestingly, the activating Fc*γ*RIIA receptor is present only in minute amounts, whereas the inhibitory Fc*γ*RIIB receptor is expressed in much higher quantity. Stimulation of basophils with IgG immune complexes, however, did not lead to their activation. Instead, an IgE-mediated response was dampened by IgG binding and this effect was even potentiated in IL-3 primed cells. This dampening effect could be a reason for symptom reduction during immunotherapy in which increased levels of allergen-specific IgG occur [80].

#### 3.1.2. Activation by Cyto-/Chemokines, Complement, and Growth Factors

*IL-3*. IL-3 was described as a key molecule in priming/activation of basophils. It is crucial for cytokine production, especially of IL-4 and IL-13 following IgE-dependent stimulation, the expression of cell surface antigens, such as CD203c and IL-1 receptor-like 1 (IL1RL1, alias ST2), and releasability of cells [81–83]. Basophils themselves release IL-3 during the late-phase response to antigens, thus, apparently creating a positive feedback loop in activation [84–86].

Furthermore, IL-3 is crucial for the expansion of basophils and an enhanced response in parasitic infection [87, 88]. Signaling through the IL-3 receptor is very similar to IgE receptor signaling and comprises the PI3-kinase/Akt/mTOR, the MEK/ERK, and the JAK/STAT pathway [65]. The overall importance and versatility of IL-3 in basophil biology will be elaborated further in the following chapters of this review.

*TSLP*. Incubation of basophils with thymic stromal lymphopoietin (TSLP) leads to a significant increase of histamine, IL-4, and IL-13 and CD203c upregulation. TSLP thus is an activation enhancer of human basophils. Neutralizing antibodies against the TSLP receptor prevent basophils from releasing IL-4 and IL-13 and inhibit CD203c upregulation. Moreover, activation by TSLP depends on an IL-3 receptor component, hence, on the IL-3-specific activation pathway, contributing to IL-3 production of basophils and high surface expression of CCR3 [89].

*IL-25 and IL-33*. During infection with parasites, viruses, or exposure to allergens, alarmin cytokines like IL-33 and IL-25 are important initiators of Th2 responses [90]. Human basophils express receptors for IL-25 (IL-17RB) and IL-33 (ST2) and priming of the cells with IL-3 as well as IgE-mediated activation upregulates receptor expression [91–94]. Basophils stimulated with IL-33 activate the NF*κ*B and p38 MAP-kinase pathways and secrete type 2 cytokines (IL-4, IL-5, IL-6, IL-9, and IL-13). Both CD203c and IL-3 receptor alpha expressions were upregulated upon activation of IL-25 or IL-33 [91, 92, 94, 95]. Besides, basophils secrete IL-25 upon IgE challenge [96] and IL-33 is able to upregulate the expression of leptin receptors on the basophil surface which can induce degranulation and cytokine synthesis [97].

*IL-18*. Although human basophils express the receptor for IL-18, a stimulation capability of this cytokine could not be observed in humans [98].

*CXCL8/IL-8*. CXCL8/IL-8 activates basophils by binding to its receptor on the cell surface in an IgE-independent manner and independent of pretreatment with IL-3. This was shown by Krieger et al. who measured the transient rise of cytosolic free calcium concentrations after activation of basophils by IL-8 [99].

*Complement*. Various other natural ligands promote basophil activation including the complement products C5a and C3a and the chemokines CCL2, CCL3, or CCL5. The complement products C3a and C5a, also called anaphylatoxins, have the ability to induce histamine and LTC4 release from human basophils. IL-3 is necessary for C3a-mediated but not for C5a-mediated histamine release, whereas LTC4 only is released in combination with IL-3 [100]. Activation of basophils with the combination of C5a and IL-3 is also able to stimulate the release of IL-4 and IL-13 [101]. The signaling pathway of C3a and C5a is initiated by G-protein-coupled receptors (GPCRs) which are also involved in the basophil activation* via* CCL2, CCL3, and CCL5 [102, 103].

*IL-5 and GM-CSF*. IL-5 and granulocyte macrophage colony-stimulating factor (GM-CSF) have been shown to contribute to basophil activation in combination with other agonists which induce histamine and LTC4 release suggesting synergistic effects. GM-CSF has a higher potency to add to histamine release compared to IL-5, whereas LTC4 production is more affected by IL-5. The underlying mechanisms relate to IL-5, rendering basophils more responsive to the anaphylatoxin C5a [104] and GM-CSF, rendering basophils more sensitive to the anaphylatoxin C3a [105]. Signaling of GM-CSF and IL-5 is very similar to that of IL-3 as they share the common signal-transducing beta-chain [106–108].

*NGF*. Human basophils even can be coactivated by nerve growth factor (NGF) as they have tyrosine protein kinase receptors (Trk) A on their cell surface. Together with IL-5, NGF can prime basophils to release LTC4 [109] and NGF can directly stimulate IL-13 secretion in human basophils [110]. Gibbs et al. investigated the effects of IL-3 and NGF on the release of histamine, IL-4, and IL-13 from human basophils and found that both had the same potential in enhancing histamine and IL-13 release and, to a lower extent, the IL-4 release in basophils primarily activated in an IgE-dependent way. In addition, high levels of NGF induced cytokine release from basophils* via* an IgE-independent route [111].

*LIR7*. Another receptor expressed on the surface of human basophils is leukocyte immunoglobulin-like receptor (LIR) 7 which is coupled to the common Fc receptor gamma chain. Monoclonal antibody-based receptor cross-linking induced the release of histamine, LTC4, and IL-4 [112].

#### 3.1.3. Activation by Bacteria-Derived Products

*Formyl-Methionine-Phenylalanine (fMLF)*. fMLF is an N-formylated tripeptide that is derived from gram-negative bacteria. It is a potent chemotactic factor for leukocytes that have the formyl peptide receptor (FPR)-1, which is the most prominent one of this receptor family, on their surface [113]. Basophils express FPR-1, 2, and 3, of which FPR-1 binds fMLF with high affinity, whereas FPR-2 is a low affinity receptor for fMLF. Upon stimulation of the cells with fMLF a signal cascade is triggered involving receptor phosphorylation, which inhibits further activation of the same receptor. The phosphorylation signal induces the release of proinflammatory mediators and triggers chemotaxis. FPR-2 and FPR-3 are mainly involved in chemotaxis to pathogen-derived molecules, for example, the* Helicobacter pylori*-derived peptide Hp_2–20_ [114].

*Muramyl Dipeptide*. Recently, the intracellular pattern recognition receptor nucleotide-binding oligomerization domain-containing protein 2 (NOD2) was identified in the cytosol of basophils [115]. NOD2 is the receptor for muramyl dipeptide, a specific structure of bacterial peptidoglycans [116]. Hence, the route* via* NOD-like receptors (NLRs) may constitute another avenue for activation by bacterial substances.

*TLR Ligands*. Pathogens from bacteria, viruses, and fungi are recognized by immune cells* via* toll-like receptors (TLRs) located on the cell surface or within cellular endosomes. So far, 13 different TLRs have been identified. TLR1, 2, 4, 5, 6, 9, and 10 mRNA and/or protein expression was identified in human basophils [117–123]. Basophils reacted to TLR2 stimulation with peptidoglycan by secreting the cytokines IL-4 and IL-13 but neither histamine nor LTC4 were released [118]. High concentrations of LPS, ligand for TLR4, enhanced CD63 surface expression and histamine release from basophils of atopic patients in combination with allergen stimulation [120]. Stimulating basophils with the TLR5 ligand flagellin enhanced IL-6 secretion from the basophils [123]. Simultaneous stimulation of the high affinity IgE receptor and TLR4 or TLR9 synergistically upregulated IL-4, CXCL8, IL-13, and CCL5 secretion [124]. Moreover, an elevated CXCL8 secretion was observed in allergic individuals upon TLR1/2 and TLR2/6 stimulation [122]. These findings suggest a connection between the pathogen-induced basophil activation and an allergic response of basophils.

#### 3.1.4. Activation by Proteases

Basophils may react to proteases* via* direct sensing of the protease activity. This circumstance earns special attention as some of the most common allergens are proteases. One famous candidate is the house dust mite allergen Der p 1 which was able to induce IL-4, IL-5, and IL-13 production from basophils independently from the presence or absence of IgE [125]. However, signaling comprises similar activation pathways compared to IgE-mediated activation. This was confirmed by a study using bone marrow-derived murine basophils which, upon direct activation by the cysteine protease papain, produced the Th2-inducing cytokines IL-4, IL-6, and TNF*α via* PI3-kinase and ERK pathways [68]. Pretreatment of such protease antigens with protease inhibitors diminished their ability to activate basophils [125].

### 3.2. Basophil Adhesion and Migration

#### 3.2.1. Adhesion

In addition to its versatile roles in all kinds of basophil responses, IL-3 also induces integrin CD11b expression and therefore is involved in the adhesion of basophils to vascular endothelial cells. GM-CSF and IL-5 share similar properties in basophil adhesion, however, with lower potential to induce integrin CD11b expression [126, 127]. Stimulation of the cells with IL-33 induced CD11b expression on basophils and enhanced eotaxin (CCL11)-directed chemotaxis* via* the basophil surface receptor CCR3 [128]. Moreover, P-selectin, CD49d, CD49e, CD49f, and CCR7 all have been identified on the basophil surface and are involved in the IL-3-mediated rolling and adhesion of basophils to endothelial cells [129].

#### 3.2.2. Migration

Upon adherence of the basophils to the cells of the vessel walls they first have to transmigrate across the endothelial cell layer followed by breaking through the basement membrane (transbasement membrane migration, TBMM) to reach the site of inflammation. Transendothelial migration (TEM) of basophils was mediated and directed mainly by eotaxin, the chemokine ligand of CCR3, and CCL5, the chemokine ligand of both CCR1 and CCR3, in freshly isolated cells. Differently, in basophils cultured for 24 hours SDF-1 induced strong migration responses* via* binding to CXCR4 on the basophil surface. Interestingly, IL-3 again has an additive effect in eotaxin-mediated TEM. Adhesion molecules involved in TEM of basophils are the *β*_2_ integrins leukocyte function antigen-1 (LFA, alias alpha-L/beta-2, or CD11a/CD18) and macrophage-1 antigen (Mac-1, alias alpha-M/beta-2, or CD11b/CD18), and the *β*_1_ integrin very late antigen 4 (VLA-4, alias alpha-4/beta-1, or CD49d/CD29). Eotaxin- or IL-3-stimulated basophils showed TEM which was mainly depending on the *β*_1_ integrins and their endothelial ligand VCAM-1 [130–133].

In contrast to TEM, chemokines had no effect on TBMM of resting basophils. However, in presence of IL-3, CXCL8 (*via* CXCR1) and CCL5 (*via* CCR1 and CCR3) were able to induce TBMM [134]. The same was observed with the lipid mediators 5-oxo-eicosatetraenoic acid and PAF. IL-3 presumably positively affects the expression of the *β*_2_ integrin and surface matrix metalloproteinase MMP-9 which cooperatively help basophils to cross the basement membrane [130, 131, 134].

Interestingly, basophils migrated upon IgE stimulation and Fc*ε*RI cross-linking. This migratory effect was even observed at minimum amounts of stimulant without triggering basophil degranulation and histamine release. The same effect was observed upon activation with Der f 2 in* Dermatophagoides farinae*-allergic donors [135].

### 3.3. Basophil Survival and Apoptosis

The foremost known cytokine prolonging basophil survival is IL-3. In trypan blue staining experiments, this cytokine was reported to prolong the lifespan of cultured basophils from maximum three days up to several weeks [136]. The survival-prolonging effect was observed even at minute amounts of IL-3 and shown to be much more potent than IL-5 or GM-CSF, two other cytokines with effect on basophil survival [127]. Moreover, stem cell factor (SCF) was shown to boost the survival effects of IL-3 [137] and also IL-25 binding to its receptor inhibits basophil apoptosis as measured by annexin V staining [91].

In contrast, human basophils express the death receptors CD95, tumor necrosis factor-related apoptosis-inducing ligand receptors (TRAIL-R)1, and TRAIL-R2 and treatment with an anti-CD95 antibody significantly increased apoptosis in basophils [98, 138]. Glucocorticoids, too, were shown to induce apoptosis in basophils and this apoptotic effect is considered responsible for their anti-inflammatory properties [139]. Antiallergic and antiasthmatic drugs also have apoptotic effects on basophils, as shown by olopatadine and theophylline treatment of purified human basophils. Besides, their apoptotic capacity even overruled survival prolonging effects of low doses of IL-3 [140].

### 3.4. Basophils as Antigen-Presenting Cells?

A number of studies performed on mouse basophils came to the conclusion that basophils are potent antigen-presenting cells. Soon after, doubts arose as others could not confirm the postulated antigen-presenting role in birch pollen-allergic patients. In coculture experiments of T cells with APCs but not with basophils T cell proliferation was determined upon allergen treatment [141] and fluorescence-labeled Bet v 1, used to detect internalization of allergen by basophils, was neither internalized, processed, nor presented [142]. The antigen-presenting effects observed in the original publication most likely were due to contamination of purified basophils with dendritic cells [143].

### 3.5. Protective Functions of Basophils

#### 3.5.1. Ectoparasites

Starting from the above-mentioned experiments with ticks performed by Trager [23] in the late 1930s, it was later shown that hosts can develop resistance to ticks by impairing engorgement and reducing viability of the parasite [144]. Infiltration of basophils to the feeding site was observed in guinea pigs and mice, and considered essential for tick resistance, as it was reduced upon basophil ablation [145, 146].

Basophils have also been shown to infiltrate sites infected by* Sarcoptes scabiei* (scabies) mites. A protective role of basophils against these mites has been deduced from a patient lacking eosinophils and basophils who was suffering from an extensive scabies manifestation [147, 148].

#### 3.5.2. Helminths

Typically, helminth infection induces activation of Th2 cells, eosinophils, basophils, mast cells, and type 2 innate lymphoid cells plus high serum IgE levels [149]. Several mouse models have been developed in which the roles of basophils in helminth infections were studied. Infection with the nematode* Nippostrongylus brasiliensis* induced sensitization of basophils which prevented secondary infection in mast cell-depleted mice [150, 151]. Another study showed that trapping of* N. brasiliensis* larvae in the skin depends on basophils which activated M2 macrophages by secreting IL-4. Migration of the larvae to the lung and intestine thus was impeded [152]. Adult* N. brasiliensis* worms were actively expelled presumably upon IgE-mediated receptor cross-linking of basophils and downstream IL-4/IL-13-dependent mechanisms [153].

A strong basophil-induced Th2 response resulted in the formation of granulomas absorbing toxic egg products and prevented surrounding tissue damage in* Schistosoma mansoni* infection [149]. In this case basophils were activated by IL-4-inducing principle of* Schistosoma* eggs/*α*1 (IPSE/*α*1). This major immunogenic trematode egg glycoprotein induces IL-4 secretion from basophils by binding nonspecifically to cell-bound IgE [154].

#### 3.5.3. Parasitic Protozoa

The role of basophils in infections by protozoa like* Leishmania*,* Toxoplasma*,* Trypanosoma*, or* Plasmodium* (malaria) is scarcely investigated.* Plasmodia* were shown to induce increased levels of histamine in the serum of infected patients [155] but elevated basophil counts were not observed [156]. In severe malaria cases basophil responsiveness, as determined by CD203c upregulation, was increased compared to mild infections [157]. In mouse studies high basophil counts protected from cerebral malaria and increased CD41 surface expression indicated basophil activation [149, 158, 159].

#### 3.5.4. Respiratory Bacteria

Basophils were postulated to contribute to antibacterial defense in the upper respiratory tract of* Haemophilus influenzae-* and* Moraxella catarrhalis*-infected patients* via* an IgD-dependent mechanism. Monoclonal IgD bound to beads was used to stimulate the release of proinflammatory and antimicrobial mediators from basophils of healthy donors, and the resulting supernatant prevented microbe replication [74, 160].

### 3.6. Basophils and Disease

#### 3.6.1. Hypersensitivity

Basophils are best known for their role in eliciting hypersensitivity reactions including immediate, late-phase, and delayed hypersensitivity reactions. Allergen cross-linking leads to release of histamine, LTC4, and PAF, key molecules of the immediate type reaction leading to typical signs of allergy ranging from itch to the life-threatening anaphylaxis [161]. Six to 12 hours after the immediate response, late-phase reactions like allergic rhinitis and asthma occur. Basophils infiltrate the affected tissues and through IL-4 secretion promote a Th2 environment [162, 163]. Also in delayed hypersensitivity reactions basophils are suggested to play a pivotal role in sustaining a Th2 milieu through IL-4 and IL-13 release [161, 164].

#### 3.6.2. Autoimmunity

Basophils may contribute to the development of lupus nephritis as patients showed higher basophil responsiveness than controls determined by CD203c upregulation. Concomitantly, serum levels of autoreactive anti-nuclear IgE correlated with disease severity. Using Lyn^−/−^ lupus nephritis mice, autoreactive IgE-activated basophils promoted a Th2 environment which led to disease aggravation* via *enhanced autoantibody production [165, 166].

#### 3.6.3. Malignancy

Increased numbers of basophils have been observed in acute and chronic myeloid leukemia, and basophilia was associated with a reduced overall survival in patients with myelodysplastic syndromes [167–169].

## 4. Basophil Activation Test

The basophil activation test or BAT is an* in vitro* method used to diagnose hypersensitivity reactions of patients. In BAT the basophils are identified by flow cytometry* via* distinct basophil surface molecules like CCR3, recently determined as one of the most robust basophil identification methods [170]. Other identification strategies use anti-CD123+/HLA-DR- or anti-CRTH2+/anti-CD3-, as mentioned above, or staining of IgE bound to Fc*ε*RI on the basophil surface [171]. Latter method, however, might potentially interfere with the activation pathway of the cells.

Next to identification, basophil activation can be measured* via* CD63 or CD203c upregulation. CD203c is a highly specific basophil molecule [172]. Both molecules are upregulated upon cross-linking of the Fc*ε*RI by allergen-IgE complexes and therefore used to detect the activation of donor basophils upon allergen stimulation in clinical diagnosis by BAT [173]. As reviewed by Hoffmann et al. [174, 175] BAT is successfully used to diagnose IgE-mediated allergies, for example, in food, hymenoptera venom, latex, and inhalant allergies. A more distinct view has to be drawn when analyzing allergies/hypersensitivities to drugs (DHRs). Suitability of BAT as a biomarker for diagnosis of immediate DHRs has been evaluated recently as a safe and, at least for some DHRs, reliable method. However, provocation testing is still of uttermost importance in clinical evaluation [176].

### 4.1. Basophil Activation Test as a Tool for Characterization of Hypoallergens

**Figure 5:**
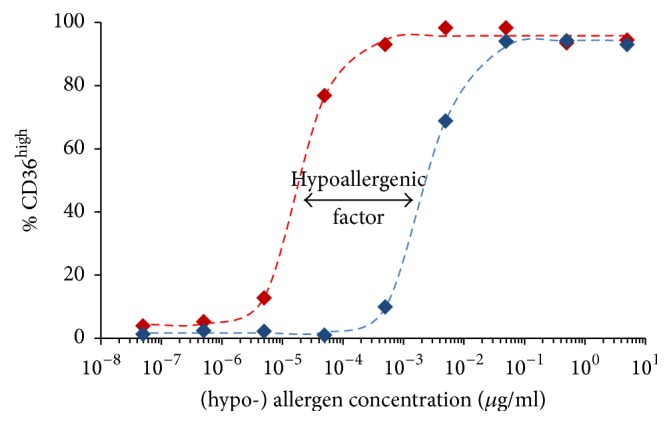
Determination of the hypoallergenic factor (here of 122) from C50 values of allergen (20.1 pg/mL, in red)* versus* hypoallergen (2.46 ng/mL, in blue). The experimentally determined activation values (⧫) have been approximated to analytically defined sigmoidal curves (broken lines) from which the C50 values were calculated [178].

In a number of reports basophils derived from allergic donors have been used for characterization of recombinant hypoallergens in the format of BAT using the activation markers CD63 or CD203c [177–185]. The decreased allergenic capacities of the hypoallergens have been evaluated in different ways though. One reliable way of calculating the hypoallergenic factor with good reproducibility is depicted in Figure 5. The hypoallergenic factor can be determined in an analytical way from the ratio of the concentrations of half-maximal basophil activation (C50 values) of allergen* versus* hypoallergen, which can be achieved by approximating the experimentally determined activation values to a sigmoidal curve using, for example, the Solver add-in of Microsoft Excel™ based on the following formula: *y* = bottom + (top − bottom)/(1 + (*x*/C50)^−slope^) [178].

An alternative approach for characterizing recombinant allergens [186, 187], hypoallergens [178, 188], allergenic extracts [178], and other allergy-related research purposes including the investigation of drug hypersensitivity reactions [189, 190] has been pursued by the use of “humanized” rat basophil leukemic cells, that were established about a decade ago and express the human high affinity IgE receptor chains [191].

## 5. Concluding Remarks

Even though they have been first described long ago, human basophils have just recently undergone an amazing development in the investigation of the functional roles in the context of the human immune system. Panels of cyto-/chemokines secreted and receptors expressed have been described. Moreover, an extensive array of other molecules including immunoglobulins, cyto-/chemokines, factors of the complement system, bacterial compounds, and growth factors have been shown to influence the activation state of basophils. A rise from being conceived as “underdogs” [192], shortly hyped as antigen-presenting cells, currently arrived at a solid and well-established role in allergy diagnosis [174] characterized the evolution of human basophils' biology.

## Figures and Tables

**Table 1:** Stimulation of basophils by different mediators.

Immunoglobulins	Cyto-/chemokines complement growth factors	Bacteria-derived products
IgE	IL-3	fMLF
IgD	TSLP	Muramyl dipeptide
sIgA	IL-25	TLR ligands
IgG	IL-33	Proteases
	IL-18	
	CXCL8/IL-8	
	Complement	
	IL-5	
	GM-CSF	
	NGF	
	LIR7	

Ig, immunoglobulin; sIg, secretory Ig; IL, interleukin; TSLP, thymic stromal lymphopoietin; CXCL, chemokine ligand; GM-CSF, granulocyte macrophage colony-stimulating factor receptor; NGF, nerve growth factor; LIR, leukocyte immunoglobulin-like receptor; fMLF, formyl-methionine-phenylalanine; TLR, toll-like receptor.
